# Is There Any Association Between Clinical and Biochemical Hyperandrogenism in Women With Female Pattern Hair Loss?

**DOI:** 10.7759/cureus.11732

**Published:** 2020-11-27

**Authors:** Samih A Odhaib, Khalil Al Hamdi, Abbas A Mansour

**Affiliations:** 1 Adult Endocrinology, Faiha Specialized Diabetes, Endocrine and Metabolism Center, College of Medicine, University of Basrah, Basrah, IRQ; 2 Dermatology, College of Medicine, University of Basrah, Basrah, IRQ; 3 Diabetes and Endocrinology, College of Medicine, University of Basrah, Basrah, IRQ

**Keywords:** alopecia, androgen excess, androgenetic alopecia, androgens, bald, hair loss, hirsutism, premenopausal women, sex hormone binding globulin, testosterone

## Abstract

Background

The exact association between clinical and biochemical hyperandrogenism (HA) is heterogeneous and cannot be ascertained, especially in normoandrogenic women.

Objectives

Evaluate any association between clinical phenotypes and biochemical parameters of HA in premenopausal women with female pattern hair loss (FPHL).

Materials and methods

A cross-sectional observational study on 362 women, who were assessed for general characteristics, the different FPHL severities by Sinclair's score, hirsutism by modified Ferriman-Gallwey (mFG) score. Evaluation for biochemical HA included total, calculated free and bioavailable testosterone (TT), free testosterone (FT), and bioavailable testosterone (BT), respectively, and dehydroepiandrosterone sulfate. The variables of clinical HA were FPHL, hirsutism, and acne.

Results

The enrolled young premenopausal women's age range was (14-47 years). Around 78% were overweight or obese women. Eighty-percent of women had a mild FPHL, with a median of three years, where 2/3 of women had a duration <3 years with no significant relationship to FPHL severity. About 73% of women had either a mild to moderate hirsutism, and around 16% had acne. The biochemical HA was confirmed in around 52% of women (n=188), who show high levels of calculated FT. The calculated BT is high in 78.5% of women (n=284). The means of HA's biochemical indicators were in their reference ranges or slightly above, with no specific change pattern with the corresponding FPHL severity. None of these parameters had a significant relationship with the severity of FPHL. The FPHL duration was not affected by any presumed variable of clinical or biochemical HA.

Conclusions

FPHL severity was associated with other clinical HA signs like hirsutism and acne, but not to HA's biochemical parameter. Other parameters, like sex hormone-binding globulin (SHBG), and BMI, had no significant relation to the FPHL severity.

## Introduction

Female pattern hair loss (FPHL) is the most common nonscarring diffuse hair loss disorder of a characteristic pattern in reproductive age women, with an uncertain relationship with androgens [1-3]. The main histopathological changes involve the hair follicles' miniaturization, with a consequent decline in hair density at different scalp regions, especially the scalp [1]. All phenotypic presentations of FPHL involve the bitemporal and vertex region in different severities. The anterior hair implantation line, frontal accentuation, and the degree of vertical baldness determine the severity of the presentation [1-3].

The study's objective was to evaluate any possible association between hyperandrogenism's clinical phenotype and biochemical parameters in premenopausal women with FPHL.

## Materials and methods

**Figure 1:**
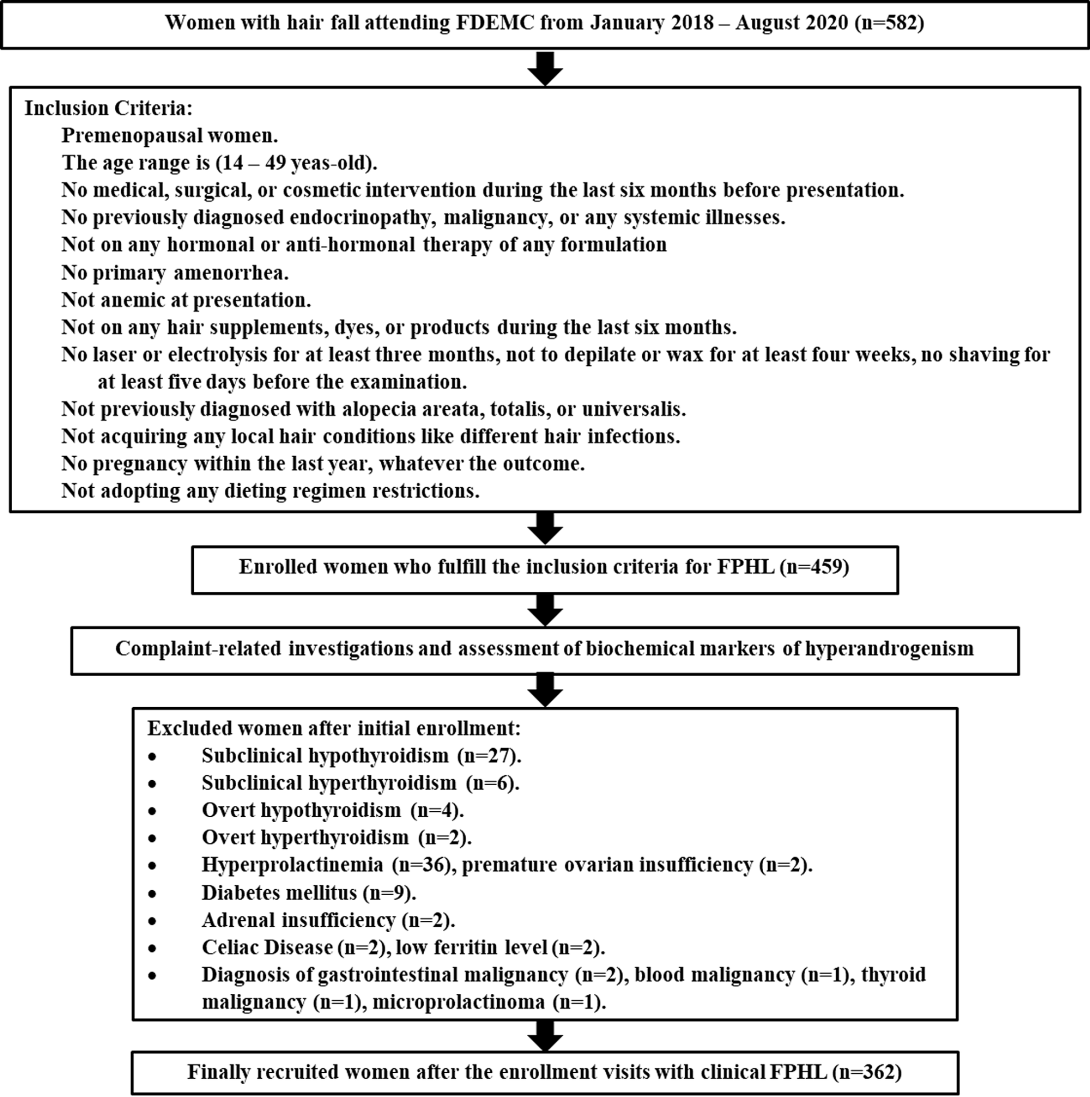
Flow chart of the inclusion and exclusion criteria

A cross-sectional observational study on (362) women with FPHL of different degrees attended Faiha Specialized Diabetes Endocrine and Metabolism Center (FDEMC), Basrah. Initially, there were 582 women with the complaint of hair fall. Enrollment and exclusion criteria are illustrated in Figure 1.

These women were either referred by gynecologists, dermatologists, internists, or self-presented directly seeking medical attention for frequent hair fall. These women were initially assessed for:

· General demographic characteristics like age, marital status, weight, and body mass index (BMI).

· Medical history in complaint-directed approach.

· Full past-medical history, gynecological, and any drug history, with relevant family history.

· Scalp examination for assessment of FPHL severity.

· Evaluation of the clinical characteristics by Sinclair's score for assessing FPHL severity modified Ferriman-Gallwey (mFG) score for hirsutism, and acne by direct clinical examination.

· Androgens evaluation included TT, sex hormone-binding globulin (SHBG), calculated FT, calculated bioavailable testosterone (BT), and DHEA-S.

Clinical HA is the presence of any cutaneous features of HA, while biochemical HA is the presence of elevated of at least one serum androgen [4].

We used Sinclair's score to assess the FPHL grades from minimal FPHL (grade 1) to severe (grade 5) FPHL [5]. The mFG score for hirsutism assessment works by the nine areas’ roles. The score of each one ranges from 1 to 4 according to the hair distribution severity. Scores ≤8/36 is the currently accepted figure for Middle-Eastern women, including Iraqi women. The hirsutism severity was graded according to (mFG) to mild (mFG score≤15), moderate (mFG score 6-25), and severe hirsutism (mFG score >25), or having no hirsutism (mFG score≤8) [6].

The variables of clinical HA which were used in this study were FPHL, hirsutism, and acne. The variables of biochemical HA were TT, FT, BT, and DHEA-S [6].

Laboratory testing

For regularly menstruating women, all hormonal investigations were performed during the first seven days of the menstrual cycle. The hormonal workup for women with amenorrhea or oligomenorrhea can be performed on any day. Early morning venous sample after at least eight hours of overnight fasting is required initially. Ten milliliters of venous blood were collected and kept in a gel tube, then centrifuged at 4100 xg by NUVE-NF 800. 

The TT, SHBG, DHEA-S were assessed using electrochemiluminescence by Roche Cobas e411 Analyzer (Germany) with standardized mean values of TT (0.52-1.60 nmol/L), for SHBG (18-86 nmol/L), and age-specific DHEA-S ranges (Appendix A.1). To calculate FT and BT, we used the method set by Vermeulen et al. [7].

Statistical analysis

We used IBM SPSS Statistics for Windows-Version 26 (IBM Corp., Armonk, NY) for the analysis of different variables. The study used the mean±standard deviation, median±standard error, and frequency (%) for data expression. For continuous variables evaluation, we compared the means using One-Way Analysis of Variables (ANOVA), while for categorical variables evaluation, the Chi-squared test was used. We used Boxplots to demonstrate the different relationships at (p ≤ 0.05) and (95% CI).

## Results

The study’s enrollment criteria were set strictly to ensure the diagnostic accuracy for the FPHL as a sole cause for hair fall in the enrolled 362 premenopausal women (Figure 1).

All the enrolled women were young premenopausal, with an age range of (14-47 years). Around 78% of the enrolled women (n=283) had a high BMI (either overweight or obese), with a mean BMI of (30.88±7.11 kg/m^2^). A mild form of FPHL was observed in 79.3% of the women (n=287), for a mean duration of (3.45±2.69 years), and about two-thirds of women had a duration less than three years, although this has no significant relationship towards the degree of the FPHL (Tables 1, 2).

**Table 1:** General characteristics of the study cohort ^a^ The continuous variables were expresses as (mean±standard deviation); ^b^ For 307 women only. Abbreviations: BT, bioavailable testosterone; FT, free testosterone; SHBG, sex hormone-binding globulins; TT, total testosterone.

Parameters		Results
Age years^a^		26.44±6.58
Age range years		14-47
Body Mass Index kg/m^2^		30.88±7.11
Women with overweight and obesity n(%)		283 (78.18)
Sinclair’s Score^a^		2±1
Female Pattern Hair Loss severity n(%)	Mild	287(79.3)
	Moderate	67(18.5)
	Severe	8(2.2)
Duration of FPHL^a^		3.45±2.69
Categories of the duration of FPHL n(%)	<3 years	231(63.8)
	≥3 years	131(36.2)
Modified Ferriman-Gallwey Score^a^		15±7
Hirsutism severity by Modified Ferriman-Gallwey Score n(%)	Mild ≤15	134(37.0)
	Moderate(16-25)	130(35.9)
	Severe >25	27(7.5)
	No hirsutism ≤8	71(19.6)
Presentation with acne n(%)		57(15.7)
TT nmol/L^a^		1.29±0.73
Women with high TT n(%)		108(29.8)
Calculated FT nmol/L^a^		0.023±0.015
Women with high calculated FT n(%)		188(51.9)
Calculated BT nmol/L^a^		0.58±0.38
Women with high calculated BT n(%)		284(78.5)
Dehydroepiandrosterone-sulfate nmol/L^a,b^		6.94±3.56
Women with high Dehydroepiandrosterone-sulfate		74(20.4)
SHBG nmol/L^a^		40.59±30.79
Women with low SHBG n(%)		61(16.9)

**Table 2:** The relation between clinical and biochemical HA variables with the severity of FPHL in 362 women ^a^ For 307 women only, representing 84.81% of women with FPHL. Abbreviations: BMI, body mass index; BT, bioavailable testosterone; DHEA-S, dehydroepiandrosterone sulfate; FPHL, female pattern hair loss; FT, free testosterone; HOMA-IR, homeostatic model assessment-insulin resistance; mFG, modified Ferriman Gallwey; SHBG, sex hormone-binding globulins; TT, total testosterone.

Parameters of hyperandrogenism		FPHL severity according to Sinclair’s score			p
		Mild (n=287)	Moderate (n=67)	Severe (n=8)	
Clinical hyperandrogenism					
Hirsutism severity according to mFG n (%)	Mild, mFG score ≤15 (n=134)	111 (38.7)	20 (29.9)	3 (37.5)	<0.001
	Moderate, mFG score between 16 - 25 (n=130)	93 (32.4)	35 (52.2)	2 (25.0)	
	Severe, mFG score > 25 (n=27)	18 (6.3)	6 (9.0)	3 (37.5)	
	No hirsutism, mFG score ≤8 (n=71)	65 (22.6)	6 (9.0)	0 (0)	
Presentation with acne n (%)	Yes (n=57)	39 (13.6)	15 (22.4)	3 (37.5)	0.048
	No (n=305)	248 (86.4)	52 (77.6)	5 (62.5)	
Biochemical hyperandrogenism					
TT	High (n=108)	89 (31.0)	16 (23.9)	3 (37.5)	0.461
	Normal (n=254)	198 (69.0	51 (76.1)	5 (62.5)	
Calculated FT	High (n=188)	154 (53.7)	28 (41.8)	6 (75.0)	0.09
	Normal (n=174)	133 (46.3)	39 (58.2)	2 (25.0)	
Calculated BT	High (n=284)	227 (79.1)	50 (74.6)	7 (87.5)	0.595
	Normal (n=78)	60 (20.9)	17 (25.4)	1 (12.5)	
DHEA-S^ b^	High (n=74)	55 (19.2)	17 (25.4)	2 (25)	0.703
	Normal (n=233)	184 (64.1)	44 (65.7)	5 (62.5)	
Other associated variables					
SHBG	Low (n=61)	47 (16.4)	14 (20.9)	0 (0)	0.600
	Normal (n=294)	234 (81.5)	52 (77.6)	8 (100)	
	High (n=7)	6 (2.1)	1 (1.5)	0 (0)	
HOMA-IR	High (n=228)	173 (60.3)	48 (71.6)	7 (87.5)	0.077
	Normal (n=134)	114 (39.7)	19 (28.4)	1 (12.5)	
BMI	High (n=283)	223 (77.7)	53 (79.1)	7 (87.5)	0.631
	Normal (n=79)	64 (22.3)	14 (20.9)	1 (12.5)	
Duration of FPHL years (mean ± SD)		3.33 ± 2.54	4.11 ± 3.28	2.25 ± 1.83	0.45

Evaluation of clinical HA was done by assessment of both hirsutism and acne. The mean mFG score was in the range of mild hirsutism, with most enrolled women (≈73%) having either a mild to moderate degree of hirsutism. A small number of women had acne and FPHL (n = 57), representing 15.7% of cases.

All the principal biochemical indicators for HA (TT, calculated FT, calculated BT, and DHEA-S) were in the reference or slightly above the reference ranges. The biochemical HA was confirmed in 51.9% of women (n = 188), who showed high levels of calculated FT. The calculated BT was high in 78.5% of the enrolled women (n=284). The mean level of SHBG was in the normal ranges, with only 61 women (16.9%) had low SHBG.

**Figure 2:**
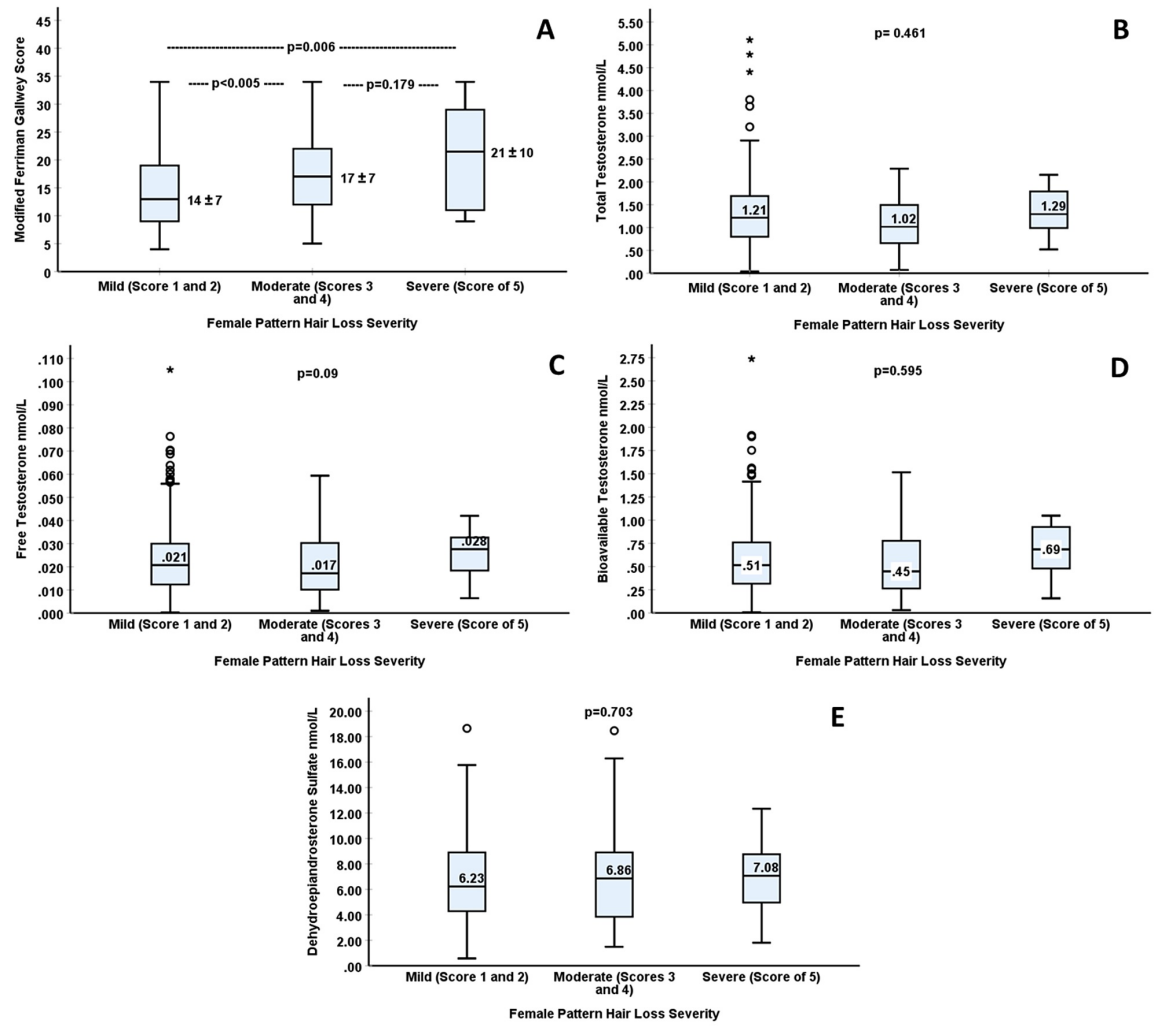
(A) Relationship between the female pattern hair loss severity by Sinclair’s score and the hirsutism score by the modified Ferriman-Gallwey score. (B-E) Relationship between the female pattern hair loss severity by Sinclair’s score with total testosterone (B), calculated free testosterone (C), calculated bioavailable testosterone (D), and dehydroepiandrosterone-sulfate (E).

Presentation with different hirsutism and acne severities had a significant relationship to the FPHL degree, although different patients fell in varying severities for clinical HA. Figure 2.A illustrated the significant step-up pattern in women with FPHL concerning the hirsutism degree, which was mirrored by the significant (within-group comparison) in One-way ANOVA. On post hoc analysis (in between groups), the difference between mild versus the moderate type of FPHL is highly significant (p < 0.001) for mild versus severe type FPHL (p = 0.006), and it was not significant (p = 0.179) between the moderate versus severe FPHL.

None of HA's biochemical parameters had a significant relationship to the FPHL severity. The mean values of the biochemical parameters of HA had no specific pattern of change with the FPHL severity (Figures 2.B-2.E). Other parameters, like SHBG and BMI, had no significant relation to the FPHL severity (Table 2). The FPHL duration was not affected by any presumed variable of clinical or biochemical HA (Table 3).

**Table 3:** Relationship between the duration and different parameters of clinical and biochemical parameters of hyperandrogenism

Parameters of hyperandrogenism		Duration (mean±standard deviation) year	p
Clinical hyperandrogenism			
Hirsutism severity by modified Ferriman Gallwey score	Mild ≤15 (n=134)	3.35±2.54	0.217
	Moderate (16 – 25) (n=130)	3.81±3.08	
	Severe >25 (n=27)	3.37±2.53	
	No hirsutism ≤8 (n=71)	3.01±2.21	
Acne	Yes (n=57)	3.45±2.56	0.988
	No (n=305)	3.45±2.72	
Biochemical hyperandrogenism			
Total Testosterone	High (n=108)	3.60±2.41	0.492
	Normal (n=254)	3.39±2.81	
Calculated Free Testosterone	High (n=188)	3.63±2.85	0.182
	Normal (n=174)	3.26±2.51	
Calculated bioavailable testosterone	High (n=284)	3.44±2.75	0.90
	Normal (n=78)	3.49±2.49	
Dehydroepiandrosterone sulfate	High (n=74)	3.70±2.88	0.479
	Normal (n=233)	3.44±2.65	
Other associated variables			
Sex Hormone-Binding Globulin	Low (n=61)	3.79±3.01	0.855
	Normal (n=294)	3.38±2.64	
	High (n=7)	3.43±2.07	
Body Mass Index	High (n=283)	3.46±2.79	0.803
	Normal (n=79)	3.44±2.33	

## Discussion

The evaluation of the association between clinical and biochemical HA in women with FPHL required two pivotal steps; the verification of association between the different clinical HA parameters and with different parameters of biochemical HA. All the possible confounders that might alter the androgen level or change the subjective evaluation of clinical HA had been excluded.

All the enrolled women were premenopausal, with an age distribution similar to other studies [4,8-11]. This study's weight distribution pattern was more prevalent towards overweight and obesity, with no significant relationship to the FPHL severity. These BMI figures are similar to other international studies [4,8,9,11,12]. However, they described different significance profiles of BMI towards different clinical HA signs due to different selection criteria from our study.

The mean Sinclair’s score of the cohort was (2±1), and represented a mild FPHL. Only 20.7% of women (n=75) had moderate to severe FPHL. The relationship between the FPHL duration and severity was not significant, even with the long mean duration of FPHL. Fattah et al. [13] described the absence of any significant association between the duration and severity of FPHL in 60 women but with a longer FPHL duration (7.56±3.5) versus (3.45±2.69) years in this study.

The other two signs of clinical HA, in addition to FPHL chosen in this study, were hirsutism and acne. It was noteworthy that not all women with FPHL had other clinical signs of HA, i.e., one-fifth of women were hirsutism-free, and more than 80% of women were acne-free. The significant correlation between FPHL and hirsutism severity was seen in Figure 2A, which described the population distribution and the different median mFG values. The post hoc analysis showed significant relationships between mild versus moderate and severe FPHL, unlike the relationship between moderate and severe types.

Hirsutism results from the interplay of the systemic and local androgens levels versus selective sensitivity and response of the pilosebaceous units' to androgens expression [14,15]. The fact that explains the limited indirect relationship between androgen level and the hirsutism severity and why the hirsutism does not always indicate hyperandrogenemia [15-17].

Considering acne as a reliable sign of clinical HA is controversial, given the previous observations for many normoandrogenic women with different acne severities, and the lack of significant direct or causal correlation between hyperandrogenemia and acne [18], which may be attributed to the selective sebum production by the pilosebaceous unit [19]. This might explain the significant association between acne and the FPHL severity in 57 women, compared to 305 women with FPHL who were acne-free, although both signs are androgen-mediated.

The FPHL duration had no significant effect on the presentation with hirsutism or acne, which could not be attributed to prolonged exposure to hyperandrogenemia, given that more than two-thirds of the cohort were normoandrogenic. The long duration may also be justified by the phenotypic heterogeneity of other clinical HA signs that urge the women to seek medical attention.

About one-third of women had high TT. We had a higher percentage of hyperandrogenism when considering the calculated FT and BT as a sole indicator of HA, with different serum-binding proteins ability [16].

There was no significant relationship between any variables of biochemical HA to FPHL severity or duration. These findings are comparable to many studies, which consider the FPHL a reliable marker for clinical HA, but not chemical HA [4,8,16].

The exact association between clinical and biochemical HA is heterogeneous [20], especially in normoandrogenic women, where genetic predisposition may play an extra role in peripheral androgen sensitization [2]. This might explain the lack of significant effect of biochemical HA parameters on the FPHL severity.

In this study, although the mean DHEA-S was normal for the enrolled age ranges, one-fifth of women had high DHEA-S. DHEA-S is a valuable marker for adrenal androgen production and activity. A normal serum DHEA-S level necessarily excludes the adrenals as HA source [21]. It has been recommended that FT be used only in patients with clinical HA with normal TT and DHEA-S [21]. DHEA-S does not bind serum proteins and does not change with any medical condition affecting them, and even it does not affect during menses [22]. Although the menstrual cycle does not affect SHBG significantly, as recommended by other authors [23].

We had four women with high DHEA-S as the sole circulating androgen, representing around 1% of women. Azziz et al. recommended against the overinterpretation of DHEA-S, if it was the sole abnormality in women with HA [24].

The SHBG level and the number of women with low SHBG did not show any significant relationship with the severity and duration of FPHL. Vexiau et al. showed a reciprocal relation between the SHBG levels and the FPHL severity [25], which might be due to the lower binding affinity at lower SHBG [8,16].

The study had a number of limitations. Not all clinical HA signs were tested; women were not examined for clitoromegaly. We acknowledge the limitation of Sinclair's score and mFG and the absence of acne's objective score. The cross-sectional design could not differentiate accidental from the causal association of the variables and the outcome and limit the results' generalizability.

## Conclusions

The FPHL was associated with other clinical HA signs like hirsutism and acne, but not to any biochemical HA parameter, i.e., there was no variable of biochemical HA that had a significant relationship to the FPHL severity. It was essential to perform an exhaustive clinical examination for women with milder phenotypes of HA because the severity was disproportional to androgen level.

## Appendices

**Table 4:** Appendix A1. Age-specific ranges of dehydroepiandrosterone sulfate, according to FDEMC central lab. FDEMC, Faiha Specialized Diabetes Endocrine and Metabolism Center.

Age ranges (years)	Concentration (nmol/L)
18 – 19	3.93 – 10.71
20 – 29	1.76 – 10.30
30 – 39	1.22 – 7.32
40 – 49	0.87 – 6.50
50 – 59	0.71 – 5.42
60 – 69	0.35 – 3.52
69 and older	0.46 – 2.44

Appendix B: Ethical Consent form for Women (in English)

·         Name of Principal Investigator: Samih Abed Odhaib

·         Name of Organization: Faiha Specialized Diabetes Endocrine and Metabolism Center

·         Name of Project: Is there any association between clinical and biochemical hyperandrogenism in women with female pattern hair loss.

This Informed Consent Form has two parts:

·         Information Sheet (to share information about the study with you).

·         Certificate of Consent (for signatures if you choose to participate)

You will be given a copy of the full Informed Consent Form

Part I: Information Sheet

Introduction

We are a study group of FDEMC. We are trying to find the association between biochemical and clinical findings regarding abnormal hormonal patterns in women who have female pattern hair loss (FPHL or androgenetic alopecia). The FPHL is frequently encountered in our practice and may cause many aspects of your quality of life to be affected, especially your perception of your body image and your self-satisfaction. You will be free to participate in this study or not, and you must keep in mind that your decision will not affect our level of medical care that was proposed to be presented to you at any time by any means. You do not have to answer now; you will have satisfactory time to agree or not. You are not obliged to give us consent today, you have all the week-long to study the consent and give us the response in your next visit a week from now, during this period you are free to contact any one of the study group to ask about any aspect you found it peculiar and need discussion. Whatever your decision will be, it will not affect our level of medical services provided to you. Keep in mind that you can withdraw your consent at any time before the complete publication of the study, and you are not obliged to give us any reasons. Again this will not affect our medical decision regarding your management by any means. You may have no questions now, but during the study, you may have questions, feel free to inform us to answer them. Is this understandable to you?

Purpose of the research

FPHL or androgenetic alopecia is a common finding in many women during their reproductive life. It affects the quality of life of women in many aspects. We want to evaluate the association of the biochemical and clinical findings of increased androgen levels. Is this association valid to be considered? Does any woman with biochemical changes of hyperandrogenism have relevant clinical changes? Is this understandable to you?

Type of Research Intervention

After you agree to participate in the study, you will be interviewed by the head of the study group (Samih Abed Odhaib), who will interview you thoroughly by history and clinical examination. He will ask about many aspects of your life that you may find embarrassing or hard to talk about like your weight, marital relation, self-perception, body image satisfaction, and sex life. He will discuss the importance of every item individually upon your request. You can take your time during the interview and you may quit answering any of the questions as you wish. This will not affect medical decisions at all.

After that, two different endocrine nurse will examine your hair by preformed scale separately using the Sinclair scale, to evaluate the degree of FPHL. They will assess the degree of your hirsutism (excessive body hair in abnormal places on women's body) using the modified Ferriman-Galway Score.

. The two endocrine nurses will be blind to each other’s results, to increase the accuracy of the findings. You will be returned to (Samih Abed Odhaib) for the interpretation of the results, and to be sent for blood investigations according to the timing of the menstrual cycle. There will be investigations in the first week of the cycle, others at the middle, and others at the end of the cycle, each will give us a clue about the menstrual pattern of yours that may be related to your FPHL or not, do you understand this or not? You need any further explanation? Additional investigations to exclude any associated illness may be sent for you after your permission, they are not additional, or for research purposes, but they will be needed in individual cases. All investigations will be needed to be drawn early in the morning in the fasting status according to the guidelines on an exact day during the menstrual cycle. You will be given an appointment within a week's duration till the results of the investigation verified by the study group. The next week you will be interviewed again to discuss the results of your investigation or to complete the battery of investigations for your complaint.

The investigations may take up to a month to be completed according to the scientific recommendations and the timing of the menstrual cycle.

During all these times you can withdraw your approval at any time, and you can request any answer for any question from the study group, and this will not affect our medical decision and the amount and the quality of medical care that will be proposed to be given to you. Do you understand? Do you have any questions till now?

All the participants will be dealt with as (registration numbers), not by names during the study progress. To keep the data as secrete as possible. All information will be registered as numbers on the SPSS data sheet directly with no names, only the registration number at the FDEMC database.

Participant Selection

You are being invited to take part in this research because we feel that your experience with FPHL can contribute much to our understanding and knowledge of local health practices, and may help us to decrease the effect of this complaint in affected women. Is this understandable to you?

Voluntary Participation

Your participation in this research is entirely voluntary. It is your choice whether to participate or not. If you choose not to participate all the services, you receive at FDEMC will continue and nothing will change.

If you decide not to take part in this research study, do you know what your options are? Do you know that you do not have to take part in this research study if you do not wish to? Do you have any questions?

You need to know:

·         We need you to participate to help us in the evaluation of the association between clinical and biochemical hyperandrogenism in women with FPHL.

·         You will not be subjected to any discussion group or focus group or any group therapy by any mean, and you will not share your information or data with anyone whether similar to your complaint or not, all the participants are blind to each other, but not to the study group.

·         We will never ask you about any personal beliefs, practices, that you are not comfortable with sharing.

·         All interviews and investigations will take place at FDEMC clinics, by the workgroups exclusively, not anyone else. All the interviews are verbal and not recorded electronically or by any means. Only data registration will take place on the SPSS datasheet by the first author (Samih Abed Odhaib) exclusively, without name identification, but only registration numbers at FDEMC. And he will be the only person that can access your data.

·         All the investigation results of the enrolled women will be downloaded by Samih Abed Odhaib exclusively. All the printed copies will be handed to you. Do you understand

Duration

The proposed time of the study is about 18 months, the study will end in July 2020. The data will be available for analysis during that period, and you will be free to participate in the study at any time, given you will fulfill the enrollment criteria at that time. On the other hand, if you accept to participate in the study you can withdraw your participation at any time before the publication of the whole study, i.e. July 2020 will be the deadline for the enrollment not for publication. Do you have any more questions?

Risks

We are asking you to share with us some very personal and confidential information, and you may feel uncomfortable talking about some of the topics. You do not have to answer any question or take part in the discussion/interview/questions if you don't wish to do so, and that is also fine. You do not have to give us any reason for not responding to any question, or for refusing to take part in the interview or questionnaire. All investigations will be done by expert personnel in the FDEMC lab who will be blind also to the study parameters. All the investigations will be done in aseptic conditions. No sample of blood will be kept for future assessment at all.

Benefits

All investigations that will be done to you are free of charge. There will be no direct financial benefit to you.

Reimbursements

You will not be provided any incentive to take part in the research. There will be no monetary reward to you by any kind.

Can you tell me if you have understood correctly the benefits that you will have if you take part in the study? Do you know if the study will pay for your travel costs and time lost, and do you know how much you will be reimbursed? Do you have any other questions?

Confidentiality

The research being done in the community may draw attention and if you participate you may be asked questions by other people in the community. We will not be sharing information about you with anyone outside of the research team. The information that we collect from this research project will be kept private. Any information about you will have a number on it instead of your name. Only the researchers will know what your number is and we will lock that information up with a lock and key. It will not be shared with or given to anyone except the first author.

Sharing the Results

Nothing that you tell us today will be shared with anybody outside the research team, and nothing will be attributed to you by name. The knowledge that we get from this research will be shared with you before it is published. Each participant will receive a summary of the results in private.

Right to Refuse or Withdraw

You do not have to take part in this research if you do not wish to do so, and choosing to participate will not affect the medical services and your evaluations in any way. You may stop participating in them at any time that you wish without any effect to the level of medical care being affected.

Who to Contact

If you have any questions, you can ask them now or later. If you wish to ask questions later, you may contact:

Samih Abed Odhaib MD.

Phone: 009647816787885.

Email: .

This proposal has been reviewed and approved by FDEMC ethical committee, which is a committee whose task it is to make sure that research participants are protected from harm. If you wish to find about more about it, contact the head of the committee:

Haider Ayad Yassin Alidrisi MD, MSc (Endocrine), Adult Endocrinologist.

Phone: 009647705021502.

Email: .

Do you know that you do not have to take part in this study if you do not wish to? You can say No if you wish to? Do you know that you can ask me questions later if you wish to? Do you know that I have given the contact details of the person who can give you more information about the study? You can ask me any more questions about any part of the research study if you wish to. Do you have any questions? 

Part II: Certificate of Consent

I have read the foregoing information, or it has been read to me. I have had the opportunity to ask questions about it and any questions I have been asked have been answered to my satisfaction. I consent voluntarily to be a participant in this study

Print Name of Participant:

Signature of Participant:

Date:      /     /

If illiterate

I have witnessed the accurate reading of the consent form to the potential participant, and the individual has had the opportunity to ask questions. I confirm that the individual has given consent freely.

Print name of witness:                                                   Thumbprint of participant

Signature of witness:

Date:       /      /  

Appendix C: Ethical Consent form for Women (in Arabic)

·         اسم الباحث الرئيسي: سامح عبد عذيب

·         مركز الفيحاء التخصصي للسكري و الغدد الصم و الايض

·         اسم المشروع: هل هناك اي ارتباط بين العلامات السريرية و البيوكيميائية لفرط منشطات الذكورة لدى النساء المصابات بتساقط الشعر الانثوي؟

هذه الاستمارة تتكون من قسمين:

·         استمارة المعلومات ( لغرض مشاركة معلومات الدراسة معك)

·         شهادة الموافقة (ليتم التوقيع عليها في حال وافقت على المشاركة)

و سوف يتم اعطاؤك نسخة من موافقتك المسبقة

القسم الاول

المقدمة

نحن مجموعة بحثية دراسية في مركز الفيحاء التخصصي للسكري و الغدد الصم و الايض, نحاول ان نوجد العلاقة بين العلامات السريرية و نمط تغير الهرمونات المختلفة لدى النساء ممن لديهن تساقط الشعر الانثوي. نصادف في عملنا هنا الكثير من حالات تساقط الشعر الانثوي, و الذي يؤثر على نوعية حياتك من عدة نواح, و خصوصا لمستوى ادراك صورتك الجسدية و تصورك لجسمك و فيما لو كنت مقتنعة به.

توطئة

لك مطلق الحرية في الاشتراك بهذه الدراسة او لا, و اعلمي ان قرارك هذا لن يؤثر البتة على مستوى الخدمة التي من المفترض ان تقدم لك من قبلنا بأي مستوى او طريقة. ليس عليك الاجابة الان, لديك الوقت الكافي للموافقة من عدمها, لست مجبرة على اعطائنا هذه الاستمارة اليوم, لديك اسبوع تقريبا لدراسة هذه الاستمارة و اعطاءنا اجابتك بعد اسبوع من الان في اثناء زيارتك القادمة, و خلال هذه الفترة لك مطلق الحرية في الاتصال بأي من اعضاء المجموعة البحثية للسؤال عن اي جزئية تجدينها غامضة و تحتاج للنقاش. و أيا كان قرارك لن يؤثر على الخدمات الطبية المقدمة لك. و تذكري دائما انك تستطيعين سحب موافقتك هذه في اي وقت قبل النشر الكامل للدراسة و ليس عليك توضيح سبب ذلك بتاتا. و ذلك ايضا لن يؤثر على القرار الطبي برعايتك باي وسيلة.

من الممكن ان لا يكون لديك سؤال الان, لكن على مدى الدراسة من الممكن ان يكون لديك, تذكري انك حرة بالاتصال باي منا لإجابته, هل هذا مفهوم لك؟

الهدف من البحث

تساقط الشعر الانثوي هو مشكلة شائعة لكثير من النساء خلال حياتهن, و هو يؤثر على نواح كثيرة من نوعية حياتهن. نحن نريد ان نقيم الارتباط بين العلامات السريرية و المتغيرات البيوكيميائية لفرط هرمون الذكورة, و فيما لو كان هذا الارتباط صالحا لكي نعتبر بخصوصه, اي فيما لو كانت اي امرأة لديها تغيرات بيوكيميائية معينة لفرط الذكورة تصاحب العلامات السريرية؟ هل هذا مفهوم لك؟

نوع التداخل البحثي

بعد موافقتك الدخول في الدراسة, سيقوم الدكتور سامح عبد عذيب و هو رئيس المجموعة البحثية, بمقابلتك و الاستفسار عن التاريخ المرضي السابق و كذلك يقوم بالفحص السريري المناسب. سوف يقوم بالاستفسار عن عدة جوانب من حياتك و التي من الممكن ان تجديها محرجة و من الصعب ان تتكلمي بها او تفصحي عنها مثل وزنك, العلاقات الزوجية و الجنسية, واقتناعك بنفسك و صورتك لجسمك. سيقوم دكتور سامح عبد عذيب بشرح اهمية كل فقرة على حدة و لديك ما يكفي من الوقت للإجابة عن الاسئلة اثناء المقابلة, و التي من الممكن ان تتركي الاجابة عن اي سؤال كيفما تشائين, و لن يؤثر ذلك على قرارنا الطبي المستقبلي البتة.

بعد هذا ستقوم اثنتان من الممرضات المتخصصات بالغدد الصماء كل على حدة و بدون معرفة نتائج الواحدة للاخرى لزيادة دقة الفحوصات السريرية, بتقييم شعرك بواسطة مقياس سنكلير,  لتقييم شدة تساقط الشعر الانثوي. كما و ستقومان بتقييم فرط نمو الشعر في اماكن مختلفة من الجسم بواسطة المقياس المعدل لفيرمان و غالواي. تؤخذ هذه النتائج الى دكتور سامح عبد عذيب الذي سيقوم بتفسير النتائج لك, و ارسال التحاليل الخاصة حسب وقت الدورة الشهرية, حيث ان هنالك تحاليل في الاسبوع الاول لها, في اثناء منتصف الدورة و اخرى قبل نهايتها, و كل من هذه التحاليل تعطينا فكرة عن نظام الدورة الشهرية لك, و فيما لو كان يؤثر على تساقط الشعر الانثوي خاصتك او لا. هل تفهمين ذلك ام تحتاجين الى توضيح اكثر؟ من الممكن ان نقوم بعمل تحاليل اضافية لك لاستبعاد علل و امراض قد تكون مرتبطة بحالتك, و طبعا ذلك يتم بعد موافقتك, و هذه التحاليل الاضافية مهمة لحالتك و ليست لأغراض بحثية, انما قد نحتاجها في حالات منفردة معينة.

كل التحاليل المذكورة نحتاج سحبها صباحا و في حالة الصيام عن الاكل و الشرب في اليوم المحدد من الدورة الشهرية حسب التعليمات و القواعد المسبقة, و سيتم اعطاؤك موعدا خلال اسبوع لاستلام نتائج التحاليل بعد ان يتم تقييمها من قبل الفريق البحثي. ستتم مقابلتك مرة اخرى الاسبوع القادم لشرح نتائج التحاليل, وفيما لو احتجت تحاليل او فحوصات اضافية او لا حسب حالتك. قد تستمر الفحوصات لشهر كامل كي تكتمل حسب القواعد و التوجيهات العلمية المسبقة و حسب مواعيد الدورة الشهرية.

خلال هذا الوقت لك مطلق الحرية لسحب موافقتك على الانخراط في الدراسة بأي وقت, كما و يمكنك طلب الاجابة عن اي استفسار من اي من اعضاء الفريق البحثي, و لن يؤثر ذلك على كمية و نوعية العناية الطبية المقدمة المفترضة لك, هل تفهمين ذلك؟ هل لديك اي سؤال لحد الان؟

كل المنخرطات بالدراسة هذه, سيتم التعامل مع بيانتهن كرقم تسجيلي و ليس كأسماء خلال المراحل المختلفة للدراسة لحفظ السرية قدر المستطاع. كل البيانات ستسجل كجزء من برنامج احصائي حسابي يسمى SPSS مباشرة كأرقام تسجيلية مركزية لمركز الفيحاء التخصصي للسكري و الغدد الصماء و الايض و بدون اسماء ايضا.

اختيار المشتركات

انت مدعوة للاشتراك في هذه الدراسة لأننا نعتقد ان تجربتك الخاصة بتساقط الشعر الانثوي من الممكن ان تساهم في تطوير فهمنا له في عملنا, و من الممكن ان يساعدنا ذلك على تقليل تأثيره على النساء المصابات بتساقط الشعر الانثوي, هل هذا مفهوم لك؟

الاشتراك الطوعي

اشتراكك في هذه الدراسة هو طوعي كليا, و هو اختيارك بالكامل فيما لو لو كنت ترومين الاشتراك او لا. و اذ لم تريدي الاشتراك فان الخدمات المقدمة لك في مركز الفيحاء التخصصي للسكري و الغدد الصم و الايض سوف تستمر و لن تتأثر باي شكل. و اذا لم تشتركي في الدراسة هل تعرفين ما هي خياراتك؟ هل تعرفين انه ليس عليك الاشتراك ما لم ترغبي بذلك؟ هل لديك اي سؤال؟

لك ان تعرفي:

·         اشتراكك سوف يساعدنا في فهم الارتباط بين العلامات السريرية و البيوكيميائية لفرط هرمون الذكورة لدى النساء المصابات بتساقط الشعر الانثوي.

·     لن يتم اخضاعك او ادخالك لأي مجموعة مناقشة او مجموعة تركيزية او اي مجموعة علاجية باي طريقة. لن تتم مشاركة بياناتك مع اي اي من السيدات اللواتي لديهن اعراض مشابهة لك. و لن يعرف اي من المشتركات بالدراسة انك مشتركة اصلا, على عكس مجموعة البحث طبعا.

·         لن نسأل ابدا عن معتقداتك او ممارساتك الشخصية و التي لن تكوني مرتاحة لمشاركتها معنا.

·     الدراسة ستجرى حصريا في مركز الفيحاء التخصصي للسكري و الغدد الصماء و الايض من قبل المجموعة البحثية حصرا. كل المقابلات ستكون شفاهية و لن تسجل الكترونيا باي شكل. سيتم ادخال البيانات في ال SPSS من قبل الدكتور سامح عبد عذيب فقط, كأرقام تسجيلية و ليست اسماء. و سيكون الشخص الوحيد من فريث البحث القادر على الدخول الى البيانات. كما و سيتم انزال التحاليل الخاصة بك من قبله ايضا.

·         سيتم تسليمك كافة النسخ المطبوعة من الفحوصات. هل تفهمين ذلك؟

مدة الدراسة

الوقت المفترض للدراسة هو 18 شهرا اي ان الدراسة ستنتهي في نهاية شهر تموز يوليو 2020. و سيتم التعامل مع جميع البيانات احصائيا بعدها. خلال هذه الفترة لك مطلق الحرية للاشتراك بالدراسة فيما لو كانت تنطبق عليك شروط الاشتراك في ذلك الوقت. و اذا قبلت الاشتراك فمن الممكن ان تسحبي اشتراكك باي وقت الى حين طبع الرسالة و نشرها, و ضعي في الحسبان ان نهاية تموز يوليو 2020 هو نهاية ادخال البيانات و ليس النشر. هل تفهمين ذلك؟

المخاطر

نحن نطلب مشاركة بعض البيانات الشخصية التي قد تكون سرية لك, و قد لا تكونين مرتاحة للحديث عن بعض منها. ليس عليك الاجابة عن اي سؤال اثناء الدراسة او عدم المشاركة في المناقشة اذا لم تريدي ذلك و بدون ذكر للأسباب هذا مقبول لنا طبعا. كل الفحوصات المختبرية ستتم بواسطة كادر مختبري مدرب تدريب عالي في مركز الفيحاء التخصصي للسكري و الغدد الصماء و الايض و الذي سيكونون غير عارفين ببياناتك الشخصية او المرضية اصلا. كل الفحوصات سوف تتم في بيئة معقمة و لن يحفظ اي من نماذج الدم او مصول الدم او البلازما الخاصة بك لغرض الفحص مستقبلا.

الفوائد

كل التحاليل و الفحوصات سوف تكون مجانية و لن تكون هنالك اي تبعات مالية عليك.

التعويضات

لن تحصلي على اي حوافز للمشاركة في الدراسة و لن تكون هنالك اي مكافأة مالية او مادية من اي نوع.

 ارجو ان تخبريني انك تفهمين بصورة صحيحة الفوائد التي تعود عليك فيما لو اشتركت بالدراسة؟ هل تعرفين اننا لن ندفع تكاليف تنقلك و الاوقات التي تقضينها. هل فهمتي ذلك؟ هل لديك اي سؤال؟

السرية

يتم عمل الدراسة في المجتمع و هذا قد يعرضك للاستفسار من قبل بعض الاشخاص في المجتمع. نحن كمجموعة بحث لن نشارك بياناتك باي طريقة خارج مجموعة البحث و سوف تظل البيانات سرية و كل البيانات الخاصة بك ستكون برقم بديلا عن اسمك. الرقم الذي سيكون معلوما فقط لدى المجموعة البحثية فقط.

مشاركة النتائج

لن تتم مشاركة اي مما تقولينه لنا لاي احد خارج المجموعة البحثية و لن يتم ربط اي من البيانات باسمك البتة. المعرفة التي سوف نستقيها من البحث سينم مشاركتها معك اولا قبل نشرها و كل مشتركة سوف تعطى ملخصا عن النتائج بسرية.

الحق بالرفض او الانسحاب

لديك الحق بعدم الاشتراك بالدراسة اذا لم ترغبي بذلك و لن يؤثر هذا على مستوى الخدمات المقدمة لك و تقييمنا لك باي وسيلة. لك الحق بإعلامنا بإيقاف مشاركتك في البحث باي وقت ترغبين.

الاتصال

اذا كان لديك اي استفسار تستطيعين الاتصال بالدكتور سامح عبد عذيب على الرقم و الايميل التاليين:

 Phone: 009647816787885.

Email: .

هذه الورقة تم تقييمها و تثبيتها من قبل لجنة اخلاقيات البحوث في مركز الفيحاء التخصصي للسكري و الغدد الصم و الايض و التي تحرص على حماية المشتركين في البحوث من اي اذى. و اذا كان لديك اي تساؤل تستطيع ان تتصل برئيس اللجنة الدكتور حيدر اياد ياسين الادريسي على الهاتف و الايميل الخاصين به 

Phone: 009647705021502.

Email: .

اخيرا هل تعرفين انك حرة في قبول المشاركة في الدراسة او لا . تستطيعين الرفض . و تستطيعين سؤالنا عن أي شيء يخصك ايضا لا عطاءك معلومات اكثر و اكثر عن الدراسة . هل لديك أي سؤال ؟

القسم الثاني :استمارة الموافقة

لقد قرات جميع المعلومات او تم قراءتها لي و كان لدي الفرصة للاستفسار عن اي سؤال و تم اجابتي بصورة وافية و انا اعلن موافقتي الطوعية لأكون جزء من المشتركات في الدراسة .

اسم المشتركة

توقيع المشتركة

تاريخ الاشتراك

و فيما لو كانت المشتركة امية

لقد شهدت على القراءة الصحيحة للمعلومات الواردة في استمارة الموافقة الخطية للمشتركة المحتملة التي كان لديها الوقت الكافي للاستفسار و سؤال الاسئلة و انا اقر انها اعطت موافقتها بصورة طوعية.

اسم الشاهد او الشاهدة و التوقيع

بصمة الابهام الايسر للمشتركة

تاريخ الاشتراك

ملاحظات الباحث لقد قرات جميع المعلومات الواردة للمشتركة المحتملة و تأكدت قدر استطاعتي انها فهمت :

·         انها تفهم الغرض من الدراسة و مختلف نواحيها

·         ان لديها الوقت لتسال الاسئلة المختلفة و قد تم اجابتها بصورة وافية من قبلنا  انها حرة بالاشتراك او لا

·         انها واعية ان كل بيانتها ستعامل بسرية مطلقة

·         لم يضغط على المشتركة باي طريقة للاشتراك في الدراسة

·         انها وقعت الاستمارة بصورة طوعية

·         نسخة من الاستمارة الموقعة سوف تسلم للمشتركة

اسم الباحث المقابل

التوقيع

التاريخ

Statement by the researcher/person taking consent

I have accurately read out the information sheet to the potential participant, and to the best of my ability made sure that the participant understands that the following will be done:

1-      She understands the goal of the study and the different aspects of it.

2-      She had all time to ask questions and was free to participate in the study or not.

3-      She was aware that all her data are confidential.

I confirm that the participant was given an opportunity to ask questions about the study, and all the questions asked by the participant have been answered correctly and to the best of my ability. I confirm that the individual has not been coerced into giving consent, and the consent has been given freely and voluntarily.

A copy of this informed consent form has been provided to the participant.

Print Name of Researcher/person taking the consent:

Signature of Researcher /person taking the consent:

Date:      /         /                                 

Appendix D: Permissions

1-    Permission to use the modified Ferriman-Gallwey score by Oxford University Press by RightsLink is available at https://s100.copyright.com/CustomerAdmin/PLF.jsp?ref=e45944c3-2d84-4a93-88fe-ee3739e69234.

2-    Permission to use Sinclair's Score by John Wiley and Sons is available at https://s100.copyright.com/CustomerAdmin/PLF.jsp?ref=263b8476-c60c-42d0-a1bb-88aed027f976.
